# The Start of a Good Innings, 50 Years of Intensive Care Medicine

**DOI:** 10.1002/wjs.70388

**Published:** 2026-05-06

**Authors:** Jonathan Oliver White, Timothy Craig Hardcastle, David Zonies, Simon Robertson, Sérgio Faria Baptista

**Affiliations:** ^1^ Intensive Care Unit 4131 Rigshospital Copenhagen Denmark; ^2^ Surgical Sciences Nelson R Mandela School of Medicine University of KwaZulu‐Natal Durban South Africa; ^3^ Trauma and Burn Services Inkosi Albert Luthuli Central Hospital Durban South Africa; ^4^ Department of Surgery UKZN Durban South Africa; ^5^ Department of Surgery University of Washington Seattle Washington USA; ^6^ Intensive Care Canberra Hospital Canberra Australian Capital Territory Australia; ^7^ Anesthesiology Department Medio Tejo Hospital Center Tomar Abrantes Portugal

## 1 Introduction

Critical care medicine is a young specialty, the conception being credited to Bjørn Ibsen during the 1950s Polio epidemic in Copenhagen. Following Ibsen's achievements in Denmark the establishment of specialized units for the critically ill spread rapidly across the world.

In 1959 in the USA the first 24‐hour ICU with senior medical staff opened at the University of Pittsburgh, the 1950s saw the first pediatric Intensive Care Unit (PICU) in Sweden. The development of “shock wards” and coronary care units followed suit in the 1960s to manage cardiac patients as well as patients with complex surgical complications.

The field of ICU was further institutionalized with the founding of the Society of Critical Care Medicine in 1970.

However, most of you reading this article will not have practiced medicine in 1970s and to reminisce on the good old days will be to relive the joy of your first pager as well as the digitalization of patient records. It is therefore necessary to understand what the baseline looked like 50 years ago. A quick google search will tell us that floppy discs were evolving, no one had a cell phone, there was no internet, Betamax (a videocassette recording device) was launched and Casio digital watches were all the rage, fast forward 50 years and we are drowned in technology that many of us could not be without but really don't understand how it works.

The authors collaborating on this work have chosen five broad areas as a framework upon which to discuss their perception of what the most important areas of advancement in Intensive Care therapy in the last 50 years have been, of course there will be bias (age adjusted) and in this subjective field there will be disagreement (hopefully).

The areas we have chosen to focus upon include technology, the use of data to improve outcomes, ICU structure, approach to patient care and advancement of professional specialization.

## 2 Technology

In the 1970s, ICU monitoring was largely limited to manual measurements of vital signs such as heart rate, blood pressure, and respiratory rate. Nurses and physicians relied heavily on clinical judgment, supported by simple electrocardiograms (ECGs) and intermittent blood gas analyses. Monitoring equipment was bulky, invasive, and often provided only snapshot information rather than continuous data.

The concept of continuous electronic monitoring began to gain traction as early cardiac monitors and mechanical ventilators became more common, marking the beginning of modern critical care monitoring. In this decade, pulmonary catheters were the mainstay of pressure monitoring and have been since, for some, relinquished to the technological graveyard [1].

The introduction of pulse oximetry in the 1980s was a milestone in improving the safety of anesthesia and critical care, around the same time capnography became available for monitoring ventilation and verifying endotracheal tube placement. Nihon Kohden developed the technology for the first ear pulse oximeter, OLV‐5100 [2], and Minolta commercialized the first finger pulse oximeter in 1977 (OXIMET MET‐1471).


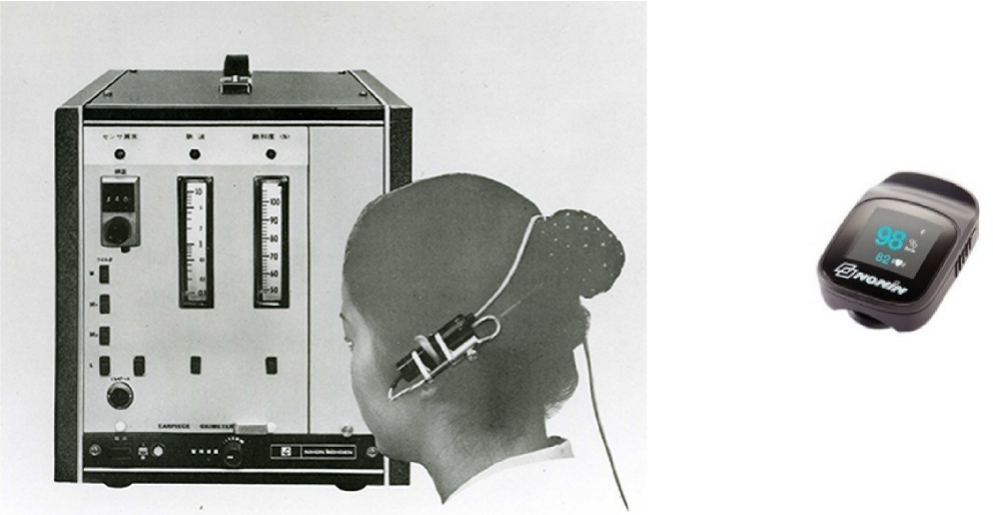


Left: First oximeter, OLV‐5100, developed by Mr. Aoyagi from Nihon Kohden in 1978. Right, a modern Bluetooth oximeter 2025.

By the 1990s, technological advances led to the development of multiparameter monitors capable of displaying continuous readings of heart rate, blood pressure, oxygen saturation (SpO_2_), and temperature which are now standard monitoring in the ICU.

Ultrasound has revolutionized the practice of ICU medicine and has its roots from 1950s, however portable ultrasound became a reality in the 1990s and its growth has been unsuppressed since. Its clinical application is beyond the scope of this review but has been described as “head‐to‐toe” diagnostics and is used to monitor everything from ICP to cardiac dynamics to pulmonary mechanics [3].

The first machines were cumbersome to use and produced images that were difficult to interpret, 50 years on and the picture quality is phenomenal and handheld devices connected by Bluetooth to mobile devices are commonplace.


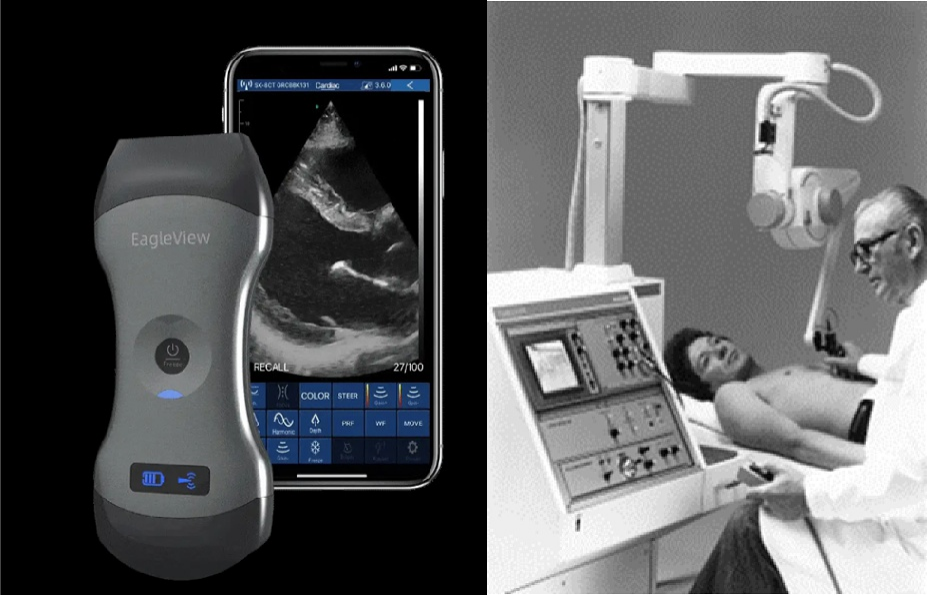


A wireless probe ultrasound scanner 2024 and a scanner from 1977.

In the 21st century, hemodynamic monitoring systems provide real‐time assessment of cardiac output, tissue perfusion, and fluid responsiveness, often using minimally invasive or noninvasive techniques. Continuous electroencephalography (EEG) and cerebral oximetry have enhanced neurological monitoring, while innovations in mechanical ventilation allow detailed tracking of lung mechanics and gas exchange.


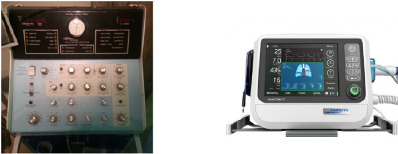


Left: Bourns BEAR 1 respirator from 1970s Right: Modern ICU respirator 2025.

The treatment of patients with renal failure deserves recognition, technological progress has led to the development of continual dialysis machines which can be operated by ICU nursing staff with minimal complications and without the presence of nephrology specialists.

Technology has also had a positive impact on airway management. In the last 10 years, we have witnessed a paradigm shift in airway management, the conception of video laryngoscopy has revolutionized airway management and improved patient safety, as adopted in the most recent Difficult Airway Society guidelines [4].

Today, artificial intelligence (AI) and predictive analytics are at the forefront of ICU monitoring innovation. Machine learning algorithms can detect subtle physiological changes before they become clinically apparent, offering early warnings for sepsis, cardiac arrest, or respiratory failure [5].

The rise of electronic health records (EHRs) and networked monitoring systems has enabled centralized observation, remote consultations, and data‐driven decision support. Tele‐ICU systems allow expert clinicians to monitor patients across multiple sites, expanding access to specialized care.

In conclusion, technological advancements have driven the practice of ICU from basic observation to intelligent, data‐integrated systems that enhance both patient safety and clinical efficiency. The ongoing integration of AI, big data, and wearable sensors promises a future where ICU care is even more precise, personalized, and proactive.

## 3 Data‐Registries and Intensive Care: A Way to Improve Outcome

Improvement in ICU‐outcome must be data‐driven to effectively classify or prognosticate outcome for critically‐ill patients [6]. Trauma registries have achieved this in polytrauma care [7, 8, 9]. ICU registries, including the Japanese, South American and ACIOS registries exist [10, 11, 12] WHO with the assistance of the World Federation of intensive and critical care societies is developing an ICU minimum data set to enable effective comparisons across the globe. Identifying gaps in the provision, capacity and clinical care of critically ill patients require standardized quality data collection.

Artificial intelligence analysis of “Big Data” enables more rapid deduction of results, potentially generating more research questions that thereby improves ICU outcome. Baseline risk assessments, administrative organization and patient management can all be addressed though data interpretation [6]. Data registries using machine learning can compare performance, measure local risk factors, optimize workflows and improve clinical management, predict adverse events, but concerns remain around clinical acceptance, information‐sharing ethics, data quality and adoption plus support in large organizations [13].

Two useful examples of the use of ICU data research to drive improved outcome though systemic change are the ORCHESTRA and ACIOS studies from Brazil and Africa, respectively [12, 14], ORCHESTRA includes data from 78 ICU's at 51 hospitals, with results showing that the presence of clinical protocols improved outcomes, both in terms of reduced mortality and with cost‐effective use of resources [14]. They also found that nurse‐to‐bed ratios did not make a huge impact on outcome, but multidisciplinary rounds were beneficial [14].

The ACIOS point prevalence study in 180 hospitals in 22 countries from Africa, where ICU resources are particularly scarce [12] demonstrated a significant incidence of critical illness and higher than expected mortality of over 20%, and demonstrated resource challenges for critical care mostly provided outside of formal ICUs, with patients receiving very basic supportive care and a severe deficit of formal ICU beds, advanced treatments (dialysis or ventilators), and the impact of HIV‐Aids. They emphasize the need for applying the 2023 World Health Assembly resolution 76.2 for integrated emergency, critical, and operative care in African ICUs [12].

Emphasizing the need for data‐driven care and registries to place ICU‐care in policy, research, and implementation focus, will improve the care of critically ill patients saving many lives from acute diseases by Global Health recognizing ICU services as universal healthcare can motivate governments and policy‐makers to bring about change.

## 4 ICU Structure: Open Versus Closed ICUs

During the past 50 years, we have been witness to an ongoing debate (battle) to document which model is most advantageous for patient outcome. The evolution of critical care medicine has been from a general hospital ward where various physicians managed their own patients (the open model) to a mature, specialized discipline with dedicated infrastructure and personnel (the closed model) [15].

In an open ICU, specialty surgical teams have full admitting rights to the ICU and continue to provide all direct patient care through the patient's ICU journey. In closed ICU models, patient care is directed by an intensivist. There also exists a middle ground, a collaborative model, where joint decisions are made between surgical teams and intensive care staff. The prevalence of the different models depends on many factors, especially geography and funding.

The benefits of open models are that there is complete continuity of care from admission to final discharge; admitting specialists have detailed knowledge and skills about their patient's conditions; and there is less risk of data loss with handover of patient care [16]. The closed ICU focusses critical care skills into the ICU and coordinates other critical care services within the hospital. In closed ICUs, treatment cohesion and practice standardization is better and there is more efficient use of ICU resources. Crucially, however, the most important question about which model produces the best patient‐centered outcomes has proven difficult to answer [17, 18].

The largest and most widely quoted meta‐analysis of 90 studies over a 30‐year period, including 444,402 medical and surgical patients showed improved ICU and hospital mortality, but no reduction in overall mortality or hospital length of stay [15].

Individual studies and more selective meta‐analyses show improved mortality at the expense of greater numbers of interventions such as arterial and central venous access [19]. The potential confounders in these studies are the levels and seniority of staffing, and the impact of universal changes in care (such as care bundles, referred to elsewhere in this article) [20, 21].

Perhaps the most important element is that the patient is either directly cared for; or seen daily by a clinician with training in intensive care. Application of these strategies has resulted in improved mortality for more than 40 years ago. This and other improved outcomes continue to be redemonstrated, even if an intensivist merely consults on patient care every day. In many parts of the world, the surgical ICU is staffed by intensivists who either have a surgical background or remain practicing surgeons. In high volume environments, this is likely the best of both worlds [22, 23].

## 5 Approach to Patient Care

There has been a dramatic reduction in mortality rates over the past 50 years related to the provision of intensive care medicine, patients whose disease states would have been deemed futile in the past are now surviving with good functional outcomes [24, 25].

The driving force of this success story are many and varied, but without a doubt the embodiment of evidence‐based medicine in our specialty has had a profound effect [26]. Evidence‐based medicine was essential for the critical appraisal of an expanding body of research and supported the development of guidelines and protocols that standardize practice and enhance the translation of evidence into bedside care, ultimately improving the quality and safety of care [27]. Numerous guidelines have been developed to address clinical practice, staffing models, and infrastructure standards in intensive care units [28]. Research has revealed barriers to guideline implementation, leading to incomplete adherence [29].

In the early 21st century, care bundles—sets of no more than five evidence‐based interventions targeting specific conditions or processes, applied using an “all‐or‐none” measurement approach—were introduced [30]. Bundles for sepsis management, ventilator care, the ABCDEF bundle, and prevention of central‐line–associated bloodstream infections have been consistently associated with improved patient outcomes [31, 32, 33, 34]. However, the effectiveness of some bundles remains inconclusive [35].

Evaluation of established ICU practices revealed that some were associated with increased morbidity and mortality, such as the use of the pulmonary artery catheter, low‐dose dopamine in renal failure, and deep sedation in ventilated patients [36]. Practice increasingly embraced a “less is more” approach, recognizing that minimizing invasiveness and avoiding overtreatment are associated with better outcomes [37].

Patient care continues to evolve with the expansion of personalized medicine, which tailors therapies to individual genomic profiles, and with advances in artificial intelligence that are increasingly support clinical decision‐making [38].

ICU practice has evolved from a disease‐centered model to a patient‐ and family‐centered approach that actively ensures the dignity and humanity of those receiving care [39].

Intensive care has evolved into a multidisciplinary team‐based approach—including, amongst others, physicians, nurses, respiratory therapists, pharmacists, rehabilitation specialists, dietitians, and a wealth of other professionals [40]. This evolution being driven by increasing patient complexity, therapeutic advancements as well as a revolution in technologies for monitoring and providing organ support the likes of which have developed exponentially over the last 50 years. Thus, each team member contributing with their unique skill set and perspectives [41]. Evidence has demonstrated that multidisciplinary care in the ICU is associated with improved outcomes, fewer adverse events, shorter length of stay, and reduced mortality [42]. Increasing emphasis has been placed on how ICU teams function, with evidence indicating that effective communication, good leadership, efficient coordination, and mutual respect are crucial determinants of performance [43].

Although the practices we use today may be regarded as obsolete in 50 years, the fundamental principles autonomy, beneficence, nonmaleficence and justice should endure.

## 6 Advancement of Surgical Critical Care in Trauma and Critical Care Education/Training: Evolution/Global Perspective

Over the past five decades, surgical critical care has evolved from a nascent concept into a distinct and indispensable subspecialty within trauma and acute care surgery. Historically, general surgeons managed critically ill patients without formalized training in intensive care. However, the increasing complexity of trauma and emergency surgical cases, coupled with advances in technology and a deeper understanding of pathophysiology, necessitated a more specialized approach [44, 45].

In North America, the formalization of surgical critical care began in the 1980s when trauma surgery matured alongside the formalization of regional trauma systems. With maturation, the American Board of Surgery recognized surgical critical care as a subspecialty. This led to the development of dedicated fellowship programs, standardized curricula, and board certification pathways. The integration of critical care into the general surgeon's portfolio has enhanced the ability to manage multi‐organ failure, sepsis, and complex postoperative complications. This has been particularly exploited to enhance multi‐disciplinary patient care in trauma centers and at academic institutions. The emergence of Acute Care Surgery as a triad encompassing trauma surgery, emergency general surgery, and surgical critical care further solidified its role [46].

In Europe, the evolution has been more heterogeneous. Countries like the UK and Germany have traditionally maintained a separation between surgical and intensive care disciplines. However, recent trends show a growing recognition of the value of surgical intensivists, especially in high‐volume trauma and transplant centers. The European Society of Intensive Care Medicine has promoted multidisciplinary training, encouraging surgeons to gain critical care competencies.

Asia presents a diverse landscape. In countries like Japan and South Korea, surgical critical care is gaining traction, particularly in urban academic centers. However, in many regions, resource limitations and workforce shortages hinder the widespread adoption of specialized training [47, 48]. Nonetheless, international collaborations and educational exchanges are fostering growth, with increasing numbers of Asian surgeons pursuing critical care fellowships abroad.

Technological advancements—such as point‐of‐care ultrasound, extracorporeal membrane oxygenation (ECMO), and advanced hemodynamic monitoring—have transformed bedside decision‐making [49]. Additionally, the COVID‐19 pandemic underscored the vital role of surgical intensivists in managing critically ill patients, accelerating the integration of critical care training into surgical education globally.

Surgical critical care has matured into a vital subspecialty, enhancing the capabilities of general surgeons in managing complex trauma and critically ill patients. While its development varies globally, the trend toward formalized training and multidisciplinary collaboration continues to strengthen its role in modern healthcare systems.

## 7 The Next 50 Years

Predicting what will happen over the next 50 years is a precarious task and many current world events which we are witnessing could not have been imagined just a few years, or even days ago, add to this the relentless technological development and combine it with geo‐political events, makes the world a much less predictable place.

So with our current knowledge and what has been achieved over the previous 50 years what could be on the horizon for intensive care?

There is no doubt that Intensive care medicine will be transformed by further advancements in technology, a shift toward personalized medicine, and a more integrated, patient‐ and family‐centered approach to care are inevitable.

Treatment will move away from generalized “labels” like sepsis and ARDS toward therapies tailored to a patient's individual molecular and cellular characteristics, leveraging detailed genomic and biomarker data collected at or before admission.

The physical ICU may become less of a single isolated unit and more of a flexible system where all hospital beds can function as ICU beds, supported by widespread telemedicine and remote monitoring (eICU systems).

Artificial Intelligence (AI) and machine learning will analyze vast datasets in real‐time to provide predictive analytics, enabling earlier detection of patient deterioration and supporting clinical decision‐making. AI will also manage logistics and administration, freeing up staff for direct patient care.

Minimally Invasive techniques and wireless monitoring systems will replace many visible wires and tubes. Smaller, more portable extracorporeal membrane oxygenation (ECMO) and CO_2_ removal (ECCO_2_R) devices may reduce the need for traditional mechanical ventilation.

Robots will become integral to routine tasks such as delivering supplies, assisting with patient mobilization, and potentially performing procedures like inserting IV lines or catheters, further reducing the physical burden on staff.

A greater emphasis on the patient's and family's experience, with unrestricted visiting hours and family involvement in care decisions. The environment will be designed for patient comfort, with automated control of light, sound, and temperature to improve sleep quality and reduce delirium.

However, caution is also needed with regard to the pace of implementation, as we have witnessed on numerous occasions the uncritical implementation of technology can have negative consequences and what can be done is not always what should be done.

A good dose of common sense and healthy skepticism is more than ever warranted for the next 50 years.

## Author Contributions

**Jonathan Oliver White:** conceptualization, writing – original draft, project administration. **Timothy Craig Hardcastle:** conceptualization, writing – review and editing, validation. **David Zonies:** validation, conceptualization, writing – review and editing. **Simon Robertson:** conceptualization, validation, writing – review and editing. **Sérgio Faria Baptista:** conceptualization, validation, writing – review and editing.

## Funding

The authors have nothing to report.

## Conflicts of Interest

The authors declare no conflicts of interest.

## Data Availability

Data sharing is not applicable to this article as no datasets were generated or analyzed during the current study.
